# District of Columbia Veterinary Medical Association

**Published:** 1895-05

**Authors:** H. W. Acheson

**Affiliations:** Secretary


Secretary.

### DISTRICT OF COLUMBIA VETERINARY MEDICAL ASSOCIATION.

A special meeting of the District of Columbia Veterinary Medical Association was held on April 18th, at the College Building of the U. S. College of Veterinary Surgeons, directly after the commencement exercises. Drs. Jacob K. Shaub and Mortimer R. Weiner were elected to membership. No other business was transacted.

H. W. Acheson,
Secretary.

